# The Diagnostic Role of Skin Manifestations in Rheumatic Diseases in Children: A Critical Review of Paediatric Vasculitis

**DOI:** 10.3390/ijms25137323

**Published:** 2024-07-03

**Authors:** Armando Di Ludovico, Marta Rinaldi, Federico Lauriola, Francesca Ciarelli, Saverio La Bella, Giulio Gualdi, Francesco Chiarelli, Kathryn Bailey, Luciana Breda

**Affiliations:** 1 Paediatric Department, University of Chieti “G. D’Annunzio”, 66100 Chieti, Italy; armandodl@outlook.com (A.D.L.);; 2 Paediatric Department, Buckinghamshire Healthcare NHS Trust, Aylesbury-Thames Valley Deanery, Oxford HP21 8AL, UK; 3 Dermatology Clinic, Department of Medicine and Aging Science, University G D’Annunzio Chieti-Pescara, 66100 Chieti, Italy; 4 Paediatric Rheumatology, Oxford University Hospitals NHS Foundation Trust, Oxford OX3 9DU, UK

**Keywords:** vasculitis, skin, paediatric

## Abstract

Skin lesions are frequently observed in children with rheumatic diseases, particularly in conditions such as IgA vasculitis (IgAV) and Kawasaki disease (KD). In paediatric vasculitis, the presence of skin lesions serves as an early indicator, emphasising the importance of timely diagnosis to prevent complications, such as cardiac or renal involvement. Conversely, autoinflammatory disorders like juvenile systemic lupus erythematosus (SLE) and juvenile dermatomyositis (DM) may manifest with cutaneous manifestations either at the onset of disease or during its progression. Identifying these skin lesions prior to the appearance of systemic symptoms offers an opportunity for early diagnosis and treatment, which has a positive influence on the outcomes. Additionally, it is noteworthy that specific rheumatological conditions, such as acute rheumatic fever (ARF) or oligoarticular or polyarticular forms of juvenile idiopathic arthritis (JIA), may exhibit occasional, but significant skin involvement, which is strongly correlated with an unfavourable prognosis. The assessment of skin is important in the holist approach to assessing patients for potentially systemic/multisystem disorder and helps distinguish discrete conditions.

## 1. Introduction

Paediatric vasculitis consists of a challenging and complex group of multisystemic conditions which often require integrated care from professionals with multiple subspecialties, including rheumatology, dermatology, cardiology, nephrology, neurology, and gastroenterology [1]. In paediatric vasculitis characterised by consistent cutaneous manifestations at the initial stages of disease (Table 1), such as IgA vasculitis (IgAV) and Kawasaki disease (KD), the presence of skin lesions serves as a valuable diagnostic tool. In IgAV, palpable purpura is a result of the inflammation of the blood vessels, and the skin lesions are often accompanied by joint pain, abdominal pain, and kidney involvement. Similarly, in KD, polymorphic and palmoplantar rashes, which include a variety of skin manifestations, like erythema, peeling, and the swelling of the hands and feet, are characteristic features. These skin lesions are typically accompanied by a fever, conjunctivitis, and changes in the lips and oral cavity. The timely identification of these skin manifestations is crucial in these rheumatic diseases to prevent potential complications. In KD, the prompt recognition of the skin rash is particularly important as it can help prevent and reduce the severity and progression of cardiac involvement, such as coronary artery aneurysms, which can lead to long-term cardiovascular complications [2,3]. In IgAV, the early detection of palpable purpura is essential to enable the early identification and appropriate treatment of renal involvement as the disease can cause the inflammation of the kidneys and potentially lead to kidney damage [2,3]. The early identification of skin lesions allows for timely intervention and appropriate management, which can significantly impact the disease outcomes and improve patient care. In summary, the presence of skin lesions in paediatric patients with vasculitis plays a crucial role in facilitating early detection, promoting optimal patient care, improving disease prognosis, and ultimately improving the overall outlook for children with these complex diseases [4].

## 2. Molecular Mechanisms in Paediatric Vasculitis

### 2.1. Inflammation and Immune Response

**Figure 1:**
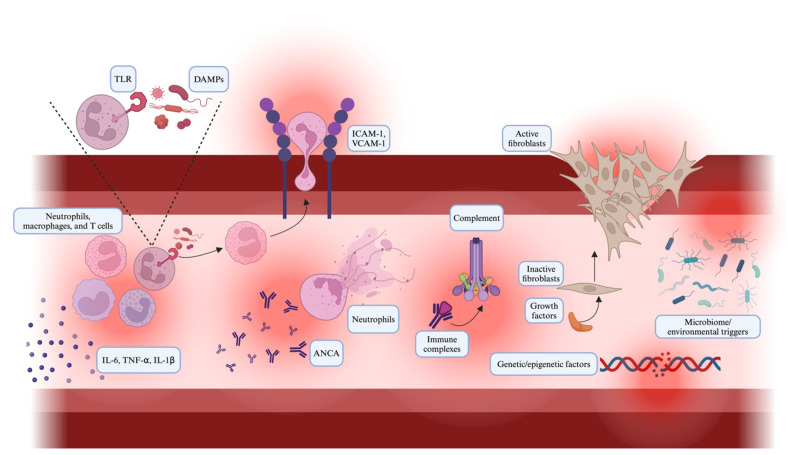
This illustration shows an inflamed blood vessel with the key mechanisms of paediatric vasculitis: pro-inflammatory cytokines (IL-6, TNF-α, and IL-1β) recruit immune cells (neutrophils, macrophages, and T cells); endothelial cells activate adhesion molecules (ICAM-1 and VCAM-1) and leukocyte transmigration; autoantibodies (ANCAs) and immune complexes trigger complement activation; growth factors recruit fibroblasts for tissue repair; and genetic and epigenetic factors, the microbiome, and environmental triggers potentially influence these mechanisms.

Paediatric vasculitis is characterised by an immune response that leads to the inflammation of the blood vessels through cytokines such as IL-6, TNF-α and IL-1β (Figure 1) [5]. These pro-inflammatory cytokines are found in a higher quantity in patients with vasculitis and act as a mechanism of the immune cells’ recruitment and activation at the site of inflammation [5]. Impaired immune tolerance mechanisms that lead to the activation of autoreactive T cells and autoantibody production, consequently escalating the inflammatory cascade, have also been raised [6]. Toll-Like Receptors (TLRs) on immune cells interact with the Pathogen-Associated Molecular Patterns (PAMPs) and Damage-Associated Molecular Patterns (DAMPs) imprinted by the pathogens and cellular damage, respectively, leading to the activation of signalling pathways that ultimately trigger an inflammatory response [7].

### 2.2. Endothelial Cell Activation

The endothelial cells that circulate in the blood vessels in patients with vasculitis may be considered as the major factor causing pathogenesis [8]. Endothelial cells synthesise activation adhesion proteins, such as ICAM-1, VCAM-1, and E-selectin, which reflects the initiation of cell migration towards the site where inflammation began [8]. These proteins are all located at the endothelial cells surface, thus aiding the adhesion and migration of leukocytes inside the vessel wall [9]. This release intensifies the vascular inflammation and adds a pathophysiological component to the clinical evidence of the disease [9]. Moreover, the endothelial cells activate, concentrate, and release pro-coagulant factors; this may lead to a pro-thrombotic status with ischemic complications [10]. The death of endothelial cells, which is observed because of inflammatory cytokines and oxidative stress, is yet another factor that leads to the breakdown of the integrity and function of the blood vessels, which are added to the list of other factors [11].

### 2.3. Autoantibody Production

ANCA-associated vasculitis involves specific autoantibodies. These antibodies are directed to antigens located in the cytoplasm of neutrophils [12]. A protein called alpha-1-antitrypsin (AAT), which is depleted in those with pANCA, binds to elastase and prevents it from destroying elastin [13]. These autoantibodies stimulate the neutrophils, causing a so-called “premature burst” of reactive oxygen species and enzymes that lead to endothelial lesions and inflammation [12]. Conversely, such immune complexes stored in the endothelial wall activate the complement system; as a consequence, the inflammatory response is enhanced in addition to the endothelial injury [14]. B cells are involved in the production of excessive antibodies, which is the case in epitope spreading; the autoantibodies continue to circulate, leading to chronic inflammation [15].

### 2.4. Tissue Damage and Repair

Vascular ischemia and the subsequent reperfusion of the smaller and more medium-sized vessels might lead to severe tissue injury. Consequently, growth factors and fibroblasts might be involved in the repair process [16]. The remodelling process includes fibrosis and a long-term change in the vessel structure, leading to worse long-term outcome [16]. The equilibrium between the destructive forces, which may be either tissue-destructive or repairing forces, and remodelling that follows the risk of chronic vascular insufficiency are critical to determine the extent of both of these functions [16]. Vascular Endothelial Growth Factor (VEGF) and the other growth factors that promote angiogenesis are a key component of tissue-repair mechanisms, as well as major pathological neovasculature genesis [17].

### 2.5. Genetic and Epigenetic Factors

Genetic predispositions have been assigned considerable meaning in the background of the susceptibility to vasculitis [18]. Modulations in the genes related to immune response, such as HLA alleles, predispose people to vasculitis or exacerbate it [18]. Epigenetic mechanisms, including DNA methylation and histone acetylation, may be involved in the pathogenesis of vasculitis stemming from changes in the gene expression of immune and endothelial cells [19]. The interpretation of these genetic and epigenetic factors may lead to the remarketing of disease mechanisms and therapeutic targets as well.

### 2.6. Microbiome and Environmental Triggers

Environmental factors like infections may contribute to an individual’s genetical predisposition to developing vasculitis [20]. The microbiome, such as the gut microbiota, is involved in the control of the immune system. Gut microbiome regulation may play a key role in autoimmune disorders like vasculitis because the a microbiome imbalance, known as dysbiosis, may trigger immune dysfunction [21]. It is important to discover the microbial signatures of vasculitis that trigger the disease and devise preventions.

### 2.7. Implications for Clinical Practice

These cellular mechanisms might significantly improve the accuracy of the therapeutic strategy for paediatric vasculitis. This is even evident when biologic agents, which are the ones used to modulate the inflammatory reaction, are utilised and, as a result, the tissue damage experienced is reduced [22]. Furthermore, the discovery of autoantibodies at early stages may be helpful for diagnosing and treating autoimmune vasculitis subtypes; where it is unique, personalised treatment may be applicable [23]. Through adding genetic, epigenetic, as well as microbiome information to clinical practice, physicians may improve their knowledge integrating all the facets of the disease process management [24]. Improved knowledge of the cellular and molecular mechanisms of vasculitis may contribute to the better treatment of patients that leads to the advancement of health outcomes [24].

## 3. Paediatric Vasculitis with Cutaneous Manifestations

### 3.1. IgA Vasculitis (IgAV)

IgA vasculitis (IgAV), previously known as Henoch–Schönlein purpura (HSP), is a type of small-vessel vasculitis and is the most common form of vasculitis in children [25]. It is characterised by widespread inflammation of the small blood vessels in the skin, gastrointestinal tract, kidneys, joints, and, in rare cases, the lungs and central nervous system (CNS) [3]. Among paediatric patients, IgA vasculitis (IgAV) is the most frequently diagnosed type of vasculitis. However, there is a lack of well-designed controlled studies on this condition, partly due to its self-limiting nature [26]. Long-term outcome data for patients with different renal features are also limited, although the prognosis for renal involvement is generally favourable, with self-resolution observed in those with minimal involvement. However, considering the risk of glomerulonephritis, or in approximately 1% of patients of the progression to end-stage renal disease, it is always appropriate to evaluate the presence of persistent proteinuria with a serrated urinary dipstick. Therefore, one of the main challenges in managing IgA vasculitis (IgAV) is achieving early and accurate diagnosis to initiate appropriate management and follow-up [27]. The presence of palpable purple lesions, known as purpura, is one of the three distinctive clinical features of the classical triad. The other two components of this triad are arthritis and gastrointestinal involvement. Purpura is considered the most crucial diagnostic factor and is a mandatory criterion for diagnosis [2]. In most cases, skin lesions are the initial manifestation of IgA vasculitis. The eruption usually starts as erythematous macular or urticarial lesions, which then progress to blanching papules, and eventually develop into palpable purpura, with diameters ranging from 2 to 10 mm. Multiple stages of skin lesions can occur simultaneously. These lesions typically appear in crops and resolve within a few days. They tend to be symmetrically distributed, primarily affecting body regions such as the ankles and lower legs in children, and the back, buttocks, and upper extremities in young children. The face, palms, soles, and mucous membranes are usually unaffected, although newborns may present with facial involvement [28]. Purpura can also be observed on the extremities, and scalp oedema is a possible manifestation of IgA vasculitis. The initial macular lesions transition to dusky, red purpuric lesions within 12 to 24 h. As the lesions fade, the purpura changes colour from red to purple, and eventually to bronze or brown. Recurrences of the lesions often occur in the same anatomical sites. In addition to purpura, hives, angioedema, and target lesions can also be seen in IgA vasculitis. Rarely, vesicular eruptions, erythema multiforme-like lesions, and haemorrhagic bullae may occur [4]. In children under two years of age, particularly those with Acute Haemorrhagic Oedema of Infancy (AHEI), the additional manifestations may include urticaria, swelling in the cranium, periorbital area, hands, and feet, as well as the presence of cockade lesions in various stages [29]. Healthcare practitioners should be aware of the range of cutaneous findings in IgAV to ensure an accurate diagnosis and guide appropriate long-term monitoring and management for renal complications and recurrent disease [30]. While the presence of leucocytoclastic vasculitis associated with IgA deposition in a skin biopsy can aid in the accurate diagnosis of IgAV, a skin biopsy is not necessary for typical lesions primarily affecting the lower limbs and buttocks. However, in cases of an atypical rash, such as extensive or diffusely distributed lesions, a skin biopsy should be performed to exclude alternative diagnoses, such as ANCA-associated vasculitis, especially in older children who may initially present with features consistent with IgAV. When conducting skin biopsies, samples should be taken from the most recent lesions [31].

### 3.2. Hypersensitivity Vasculitis

Hypersensitivity vasculitis shares significant similarities with IgA vasculitis (IgAV), particularly in terms of the affected blood vessels and clinical presentation. However, hypersensitivity vasculitis exhibits a broader range of clinical manifestations [3]. In addition to skin involvement, vascular hypersensitivity is commonly characterised by symptoms such as arthralgia and transient myalgia, oligoarthritis, skin nodules, and ulcerations. It is important to note that eosinophilia is observed in more than 5% of cases [32]. This disease can be triggered by drugs such as penicillin, sulfamide, cefaclor, anti-thyroid drugs, and occasionally other medications, although this is rare [32].

### 3.3. Kawasaki Disease (KD)

Kawasaki disease (KD) is the second-most-common childhood vasculitis. It is an acute febrile illness characterised by the acute inflammation of medium-sized arteries, making it the second-most-common vasculitis in paediatric patients. KD carries the risk of developing coronary artery aneurysms (CAAs) and can lead to sudden mortality. The timely initiation of treatment significantly reduces the formation of CAAs, decreasing the risk from 20–25% to 3–5% [2]. Currently, there is no definitive test or characteristic clinical features for the diagnosis of Kawasaki disease (KD), so the detection of a variety of clinical findings and laboratory features is necessary considering KD diagnosis. The clinical progression of Kawasaki disease (KD) is traditionally divided into three stages: acute, subacute, and convalescent. The acute stage begins with a sudden onset of a fever and usually lasts from 7 to 14 days. The fever in KD often follows a high-spiking pattern, with peak temperatures reaching 102–104 °F (39–40 °C) or higher, and it tends to be unresponsive to antibiotic treatment [2]. If left untreated, the fever may persist for three to four weeks. The Administration of Intravenous Immunoglobulin (IVIG) is typically associated with the resolution of the fever within 36 h. During the acute stage, a polymorphic rash, characterised by various types of skin lesions, commonly appears concurrently with the fever, and subsequently resolves [32]. The polymorphic rash in Kawasaki disease (KD) is primarily seen on the trunk, but can also spread to the face, extremities, and perineum. It is characterised by a lack of itchiness and presents in various forms, including macular, papular, scarlet-like, and purple lesions [32]. Additionally, the characteristic features of KD include red-violet erythematous lesions and oedema on the palms of the hands and soles of the feet. The peeling of the skin typically begins at the borders of the fingernails. Perineal erythema may occur and may be followed by peeling [2]. It is widely recognised that KD also affects the mucous membranes, leading to the inflammation of the lips and oropharynx, as well as an erythematous tongue with prominent papillae, often referred to as “strawberry tongue”. In Kawasaki disease (KD), the lips exhibit a reddened appearance with prominent swelling and vertical actively bleeding fissures. In the absence of palpebral conjunctival involvement, bilateral hyperaemia can be observed in the conjunctiva of the bulbar region, referred to as non-purulent conjunctivitis, with no exudate or redness alongside conjunctiva [2]. The subacute phase follows the resolution of the fever and lasts from 4 to 6 weeks. During this stage, the desquamation of the extremities is commonly seen, along with thrombocytosis (with a potential platelet count exceeding 1 million/L). There is a high risk of developing coronary artery aneurysms (CAAs) during this phase, with young age associated with increased risk of severe disease. Approximately from 20% to 40% of patients may experience arthralgia or arthritis, particularly affecting major joints such as the knees or ankles. The other manifestations of the subacute phase may include persistent irritability, the loss of appetite, and conjunctival inflammation. A prolonged duration of fever exceeding 2 to 3 weeks might be considered as an indication of persistent KD. A poor prognosis is considered in cases of the persistence of a fever, considering their association with a higher risk of cardiac complications [33]. Cases with a persistent fever are associated with a higher risk of cardiac complications, indicating a poor prognosis [33]. The convalescent phase is characterised by the complete resolution of clinical symptoms, typically occurring within three months from the initial presentation. This stage begins when acute phase reactants and other laboratory abnormalities return to the normal levels. Rarely, from about one to two months after the onset of fever, deep transverse fissures known as Beau lines may appear on the fingernails [33]. Cardiac abnormalities may persist during the convalescent period, but approximately 60% of cases with smaller coronary artery aneurysms (CAAs) tend to resolve spontaneously. On the other hand, larger coronary artery aneurysms (CAAs) have the potential to further expand and increase the risk of myocardial infarction (MI). The occurrence of newly detected aneurysms after 8 weeks of illness is rare in patients who had normal echocardiograms prior to their illness. Giant aneurysms may occur, and also aneurysms in non-coronary vessels, particularly when giant aneurysms occur [34]. Treatment with high-dose IVIG is recommended for children with a fever of 4 days’ duration and four of five classic clinical criteria, as well as for those with fewer clinical criteria in whom coronary abnormalities are noted via echocardiogram. There is a lower threshold to consider the treatment of younger children with or without cardiac manifestations and incomplete clinical features [2].

### 3.4. Behçet’s Disease

Behçet’s disease (BD) is an uncommon vasculitic disorder characterised by recurring oral aphthous ulcers, genital ulcers, uveitis, and various systemic manifestations [35]. A study has demonstrated that approximately 75% of individuals diagnosed with Behçet’s disease present with diverse cutaneous manifestations, including acneiform lesions, nodules, and erythema nodosum [36]. Painful oral lesions, specifically aphthous or herpetiform lesions, serve as the primary diagnostic criterion and represent the initial manifestation in 70% of cases. These oral lesions typically manifest in keratinised areas of the oropharynx, while the nonkeratinised surfaces of the dorsal tongue, molars, and hard palate are generally unaffected. The lesions observed in Behçet’s disease may bear resemblance to those seen in various other conditions, but they often exhibit a notable tendency for recurrence, surpassing the frequency specified in the International Study Group criteria (typically more than five times per year compared to the stipulated three times per year). Furthermore, these lesions tend to appear in clusters, often presenting with more than six lesions simultaneously [36]. Skin lesions may be observed in the genital regions of both males and females. In the male population, the prevalence of scrotal involvement is higher, although it is worth noting that lesions on the penile shaft can also occur. In females, the labial region is the primary site of involvement, while lesions in the vaginal and perineal areas are less common. Ulcerations occurring in the genital region in Behçet’s disease often heal with the formation of scar tissue, which can lead to increased levels of pain, particularly in males. The occurrence of ulceration in women may be correlated with the menstrual cycle [37]. Painful and erythematous nodules resembling erythema nodosum are more prevalent in the lower extremities of female patients. These nodules typically resolve within two to three weeks, but tend to recur. Erythema nodosum has been found to be moderately associated with the severity of Behçet’s disease [38]. Acneiform papulopustular lesions are more common in males and tend to appear on the trunk and extremities, although they can occur on any part of the body [18]. Only a small percentage (3%) of patients, particularly those from Bangladesh, develop extragenital ulcerations in Behçet’s disease, which tend to heal with scarring. These extragenital ulcerations typically involve areas such as the groin, axillae, neck, breasts, and the interdigital tissue of the feet. The occurrence of a positive pathergy test, which is a non-specific hypersensitivity skin reaction induced by a needle prick, is more commonly observed in Turkish and Japanese populations, as well as among individuals with ophthalmic and neurological symptoms [37].

### 3.5. Polyarteritis Nodosa

Polyarteritis Nodosa (PAN) is defined as a necrotising inflammation of the small-to-medium-sized arteries that is not associated with antineutrophil cytoplasmic antibodies (ANCAs) [39] and represents a relatively rare vasculitis in the paediatric population. According to several studies, including a multicentre survey of 110 children conducted by Ozen et al. (2004), the peak age of onset for PAN in children is typically around 9 years. The skin, muscles, kidneys, and gastrointestinal tract are the most frequently affected sites, while the involvement of the heart, central nervous system (CNS), and peripheral nervous system (PNS) is less common. The inflammation of the small arteries leads to the development of skin rashes, including livedo reticularis, purpura, necrosis, and potentially, digital gangrene. Additionally, the presence of painful subcutaneous nodules along the affected vessels is a characteristic feature [1]. Cutaneous PAN (cPAN) is recognised as a separate entity as it is characterised by the mere involvement of the skin, with no major organ system involvement. The cutaneous symptoms are similar to systemic PAN, but a mild fever, muscle and joint pain, and peripheral nervous system involvement may also occur. Most children have a chronic and relapsing benign course. Definitive diagnosis is made using histopathologic evidence of the necrotizing inflammation of the medium- and small-sized arteries. Although identical skin lesions are common in systemic PAN, cutaneous PAN should be considered a separate disease and distinguished from systemic PAN, as the clinical courses and management of these conditions differ from each other. Systemic PAN is vasculitis that causes the destructive inflammation of the medium-sized muscular arteries of multiple systems including the liver, kidneys, heart, lungs, gastrointestinal tract, and musculoskeletal and nervous systems. Systemic PAN is a potentially life-threatening form of vasculitis, whereas cutaneous PAN usually runs a chronic, but relatively benign course [40].

### 3.6. Takayasu Arteritis

Takayasu arteritis (TA) is an idiopathic, granulomatous, large-vessel arteritis that primarily affects the aorta, its major branch arteries, and, less frequently, the pulmonary arteries [41]. Granulomatous inflammation of major arteries is the most common cause of this condition, making it the third most common cause of vasculitis in children and adolescents [42]. Inflammation and endothelial damage lead to arterial wall thickening, thrombus development, and stenotic and occlusive lesions, while the deterioration of the muscular and elastic layers of the blood vessel result in dilatation and aneurysms [43]. The constitutional symptoms are more prevalent in children than in adults, with hypertension being the most common symptom. The other common symptoms include headaches, fever, dyspnoea, weight loss, vomiting, and musculoskeletal symptoms, such as myalgia, arthralgia, or arthritis [44]. Cutaneous manifestations are present in 2.8–28% of patients [45]. Livedo reticularis, purpura, erythema nodosum, subcutaneous oedema, urticaria, digital gangrene, and ulcers are all possible skin manifestations of this condition. Ulcerations may resemble pyoderma gangrenosum in certain cases [46]. Although it is rare in children, TA has been reported in some cases. A retrospective study in the United Kingdom found that the median age of diagnosis was 11.8 years, with cutaneous abnormalities observed in two patients [47]. There have been a few reported cases of chronic recurrent multifocal osteomyelitis (CRMO) and Sweet’s syndrome with post-inflammatory elastolysis and TA, mostly in children [48]. The classification criteria for childhood TA include the presence of angiographic abnormalities and at least one of the following features: a decreased/absent peripheral artery pulse(s) or claudication of the extremities, a blood pressure difference exceeding 10 mmHg, bruits over the aorta or its major branches, and hypertension (related to childhood normative data) [49]. The erythrocyte sedimentation rate (ESR) and C-reactive protein (CRP) level are commonly used acute phase markers to monitor disease progression [50]. Elevated CRP levels have been associated with thrombotic events in several studies [51]. The active periods of the disease may also be accompanied by normocytic and normochromic anaemia, leucocytosis, thrombocytosis, and high levels of amyloid A and fibrinogen in the serum [52]. The management of large-vessel vasculitis includes the early initiation of corticosteroid therapy to induce remission, the use of immunosuppressive medications as adjunctive therapy, and monitoring therapy with inflammatory markers [53]. Blood pressure control is an important intervention, and in children, beta-adrenergic blockers, calcium channel blockers, diuretics, and angiotensin-converting enzyme (ACE) inhibitors have been used.

### 3.7. ANCA-Associated Vasculitis

ANCA-associated vasculitides (AAVs) are a category of systemic vasculitis characterised by necrotizing inflammation, primarily affecting the small blood vessels. They are linked to the presence of antineutrophil cytoplasmic antibodies (ANCAs), typically directed against myeloperoxidase (MPO) or proteinase 3 (PR3) [39]. This category encompasses Granulomatosis with Polyangiitis (GPA), Microscopic Polyangiitis (MPA), and Eosinophilic Granulomatosis with Polyangiitis (EGPA). The information on epidemiology is limited. The most extensive collection of data on paediatric AAV to date stem from a multicentre study in North America, encompassing 231 patients with MPA and GPA, as outlined by Cabral and colleagues. Notably, the study revealed that the children diagnosed with MPA were younger, with a median age of 12 years, compared to those diagnosed with GPA, whose median age was 14 years. EGPA is the least common of the three subtypes [54]. The aetiology of AAV is not entirely known. Some theories include autoimmune reactions, hypersensitivity to unknown antigens, and the sensitization of the respiratory tract to bacterial pathogens. Neutrophils activated by both infectious agents and ANCA play a central role in initiating damage to the endothelial cells and tissues. This process leads to the inflammation of the vessel wall, and in the case of GPA, the formation of granulomas [55]. In paediatric AAV, more than 50% of patients commonly present with constitutional symptoms, including a fever, fatigue, anorexia, and weight loss. These non-specific findings may precede systemic organ manifestations, leading to potential delays in AAV diagnosis, especially in cases with mild renal or otolaryngological symptoms [56]. In MPA, renal disease is the primary presentation, followed by systemic features, musculoskeletal, cutaneous, lower respiratory tract, and gastrointestinal involvement. GPA typically manifests with ear–nose–throat (ENT) symptoms, followed by constitutional symptoms and renal, lower respiratory tract, musculoskeletal, and cutaneous involvement, and the loss of hearing [57]. Skin lesions, musculoskeletal symptoms, and neurological involvement are shared by both GPA and MPA in paediatric and adult-onset AAV. Notably, subglottic stenosis is more frequent in childhood-onset AAV, while cardiovascular manifestations are less common in paediatric cases. The limited data on paediatric EGPA suggest a presentation with poorly controlled asthma, eosinophilia, sinusitis, nasal polyps, lung infiltrates, cardiomyopathy, skin lesions, and gastrointestinal involvement [58]. Skin involvement was prevalent in 48% of the patients, with palpable purpura and/or petechial rash being most common, occurring in 31% of MPA and 27% of GPA cases. The less frequent skin findings included subcutaneous nodules, infarctions, livedo reticularis, Raynaud’s phenomenon, and subcutaneous swelling. Mucous membrane or eye involvement was reported in 40% of all the patients, featuring red and/or painful eye conditions. Proptosis with a retro-orbital mass was observed in three GPA patients, and one MPA patient exhibited retinal issues. Oral ulcers were relatively uncommon, presenting in 4% of the MPA cases and 15% of the GPA cases [54]. Blood tests typically show an increase in non-specific inflammation markers (ESR and CRP) and ANCA titres. Diagnosis can be supported by a tissue biopsy. The long-term outlook for AAV remains less-than-optimal. Recent meta-analyses revealed a 2.7-fold higher overall mortality rate in AAV compared to that of the general population, with a specific risk of 2.63 for GPA. Patients with PR3-positive GPA and better initial renal function are more prone to relapses. The causes of mortality in AAV encompass uncontrolled active disease, adverse events, infection, and cardiovascular complications. AAV also carries an elevated relative risk for cardiovascular events, ischaemic heart disease, and cerebrovascular accidents [59].

### 3.8. Juvenile Systemic Lupus Erythematous (jSLE)

Juvenile systemic lupus erythematosus (jSLE) is a rheumatic disorder characterised by the presence of autoantibodies targeting self-antigens, the formation of immune complexes, and immune dysregulation. These factors collectively contribute to the damage of various organs within the body, including the kidneys, epidermis, blood cells, and nervous system. Skin involvement is a variable findings that may manifest either at the onset and during the disease’s course [34].

Approximately 70–80% of patients present a malar, or butterfly rash localised on the cheekbones and nasal bridge, sparing the nasolabial fold [4]. The other types of frequently observed rashes include vasculitic macular eruptions, which tend to occur on the distal extremities and in the subungual region, showing visible microinfarcts resulting from small vessel vasculitis. Purpura, characterised by the purple discoloration of the skin due to bleeding underneath, is also commonly observed. Livedo reticularis, a condition characterised by a net-like pattern on the skin, is often associated with the presence of antiphospholipid antibodies. Alopecia, typically affecting the frontal or hairline areas, is an additional notable finding. Lastly, Raynaud’s phenomenon, characterised by sequential colour changes in the fingers, is frequently observed as well [35]. Less commonly, skin rashes consist of subacute psoriasiform or annular lesions, often linked to anti-Ro antibodies, and bullous lesions, which are rare, but life-threatening due to them covering extensive areas of the skin and oral mucosa and leading to compromised skin integrity and airway obstruction [35]. The diagnostic skin findings include a discoid rash, which is less prevalent among children, a photosensitive rash, and alterations in mucous membranes ranging from vasculitic erythema to the presence of large, deep ulcers on the palate and nasal mucosa [36].

The face, extremities, and trunk may experience various types of lesion, which can manifest as either temporary or long-lasting hyperpigmentation. A noteworthy symptom frequently observed in a significant proportion of cases is the presence of persistence haemorrhagic and ecchymotic lesions on the lower extremities in different stages of progression. The occurrence of these lesions is considered to be more prevalent in patients undergoing steroid therapy and those with disease-specific vascular damage [37]. The symptoms of vasculitis are observed in more than fifty percent of cases and can lead to the development of persistent skin ulcers. In approximately 20% of cases involving juvenile systemic lupus erythematosus (jSLE), there is a likelihood of experiencing recurring ulcers in the oral and vulvar regions. This disease exhibits photosensitivity, which exacerbates both the cutaneous and systemic manifestations [7].

### 3.9. Cutaneous Lupus (CL)

Chronic cutaneous lupus (CL) is infrequently observed in paediatric patients, and it generally manifests as a localised condition without systemic involvement or progression. Discoid lesions have an impact on the face, scalp, extremities, and neck. The observed lesion present a persistent localised redness, accompanied by the shedding of skin cells, the potential development of dilated blood vessels, and eventual progression towards either the thinning of the skin or increased pigmentation [38].

### 3.10. Neonatal Systemic Lupus Erythematous

Skin manifestations are observed in approximately 30–50% of neonatal systemic lupus erythematosus (SLE) cases. Neonatal SLE refers to the occurrence of SLE in infants born to mothers with lupus. This condition arises as a result of the transfer of autoantibodies, specifically anti-Ro IgG, from the mother to the foetus during the gestational period between the 12th and 16th weeks. This dermatological condition is characterised by a photosensitive erythematosus–squamose rash during the perinatal period and resolves by the sixth month. The condition has an impact on various parts of the body, including the face, trunk, extremities, and scalp [39].

### 3.11. Juvenile Dermatomyositis (JDM)

Juvenile dermatomyositis (JDM) is an illness characterised by distinct skin alterations, accompanied by prolonged muscle weakness or increased levels of muscle enzymes that persist for more than six months [40]. Approximately 25% of patients may develop clinically significant myositis. Juvenile dermatomyositis (JDM) primarily impacts the skin and musculature, and there are several cutaneous manifestations that tend to be related to this condition [41]. The predominant clinical manifestation is the presence of oedema in the orbital and periorbital regions, accompanied by the purplish discoloration of the upper eyelid and the development of telangiectasis along the margins of the eyelids. Notably, these symptoms may persist for an extended duration, even after the resolution of the underlying disease. The butterfly rash commonly observed in individuals bears a resemblance to the rash associated with systemic lupus erythematosus (SLE) [42].

The presence of maculopapular erythematosus, violaceous, shiny, elevated papules, and plaques on the extensive metacarpo-falangeal and interphalangeal surfaces of the hands are distinctive symptoms known as Gottrons’ signs. Similar lesions may also be observed on the elbows, knees, and trunk. Exposure to sunlight has been found to worsen skin conditions [42].

It is possible to observe the presence of nailfold telangiectasias, periungual erythema, poikiloderma, lichenification, and psoriasiform dermatitis. Hypertrophic and irregular cuticles may be observed in conjunction with periungual erythema. Children who receive inadequate treatment present persistent nailfold abnormalities that suggest the ongoing activity of skin disease, without any associated muscle involvement. The presence of diffuse vasculopathy has been observed to potentially correlate with vasomotor instability, including manifestations such as Raynaud’s phenomenon, livedo reticularis, or vascular infarctions occurring on the medial canthus of the eyelids [43].

A mechanic’s hands may show the presence of hyperkeratosis and desquamation of the skin on the lateral and palmar aspects of the fingers. The presence of myositis-specific autoantibodies and interstitial lung disease are commonly observed in mechanics [44].

Symmetrical proximal muscle atrophy, which manifests as the degeneration of the deltoids, quadriceps, or both, holds significant clinical relevance within the context of juvenile dermatomyositis (JDM). The infants in another study presented a Gower manoeuvre, which refers to their recourse to their upper limbs to raise themselves from a supine position to an upright stance [44].

The occurrence of calcinosis, a manifestation of dystrophic calcification characterised by normal serum calcium and phosphorus levels, is observed in approximately 20–40% of patients with juvenile dermatomyositis (JDM). The deposits manifest as dense, ivory, or pale-toned nodules located on prominent areas of bone, containing high concentrations of calcium hydroxyapatite, osteopontin, osteonectin, and bone sialoprotein. The distressing complication known as calcinosis, which is observed in the more severe manifestations of this condition, has the potential to impact not only the fibrous bands, but also the adipose and subcutaneous tissues, resulting in the development of painful ulcers characterised by the discharge of calcium salts. The preferred sites for stress accumulation include areas such as the elbows, knees, and buttocks [45].

### 3.12. Localised Scleroderma

Localised scleroderma (LS) is not uncommon in paediatric patients, conversely to systemic scleroderma (SS). LS is classified into three distinct subtypes, namely morphea, generalised morphea, and linear scleroderma [16].

Morphea is the localised area of skin with oedema that is warm, encircled by a hyperemic-violaceous border with distinct central region that subsequently developing into a rigid, oval-shaped lesion measuring up to 20 cm in diameter. Generalised morphea is characterised by an elevated number of spread lesions [17].

Linear scleroderma is characterised by an expanded region of densely fibrotic and pigmented skin, commonly observed on the extremities or facial region. The course of illness shows up as a fibrotic condition that involves various part of the body, including the integumentary and musculoskeletal systems. This condition leads to dysmetries and limbs contractures, as well as facial deformities [17]. The lesions may spontaneously evolve or fading. Currently, no evidence is available to support the effectiveness of any treatment in preventing the progression of the disease, but we are excepting some uncontrolled studies showing the potential effectiveness demonstrated by the administration of penicillamine or a combination therapy with cortisone boles and methotrexate [18].

### 3.13. Sjogren Syndrome

Sjogren’s disease (SD) is a chronic, systemic autoimmune disorder characterised by the immune-mediated destruction of the exocrine glands. Around 4 million individuals in the United States are affected by this condition. Historically, paediatric SD (pedSD) was considered as having a relatively infrequent occurrence. Nevertheless, there has been a notable rise in the number of reported cases. The term “Primary” Sjögren’s syndrome (SD) is used to describe cases of SD that occur independently of any coexisting autoimmune diseases, although it is noteworthy that the presence of such comorbidities is frequently observed. In these cases, the primary manifestation of SD often involves mucocutaneous symptoms, commonly referred to as “sicca syndrome”, which includes a dry mouth (xerostomia) and dry eyes (xerophthalmia) [23].

## 4. Discussion

The classification criteria endorsed by the international rheumatology scientific organizations include a wide range of cutaneous lesions [28,34,60,61], highlighting their significant role in classifying inflammatory rheumatic diseases. Cutaneous lesions often appear early in the disease process, allowing for early diagnosis before the onset of systemic symptoms. Early detection enables the timely initiation of therapeutic interventions, reducing the risk of severe complications in untreated patients. Furthermore, cutaneous manifestations in numerous inflammatory rheumatic diseases can cause considerable disability and significantly affect the health-related quality of life of patients. As a result, these skin lesions require particular attention from healthcare providers and play a crucial role in determining the effectiveness of therapeutic interventions [62]. To minimise the risk of misdiagnosis, which can have serious implications for patients’ well-being, it is crucial to have a thorough understanding of the cutaneous manifestations. Achieving more accurate diagnoses, and subsequently tailoring personalised therapy are crucial in the management of rheumatic conditions. This is particularly important in paediatric patients, where the accurate characterisation and assessment of cutaneous abnormalities can enhance the efficacy of therapeutic interventions in clinical trials [4]. For systemic lupus erythematosus (SLE), cutaneous manifestations, such as a malar rash and discoid lesions, are often the first indicators of disease and are associated with severe manifestations like lupus nephritis. Juvenile dermatomyositis (JDM) presents with a heliotrope rash and Gottron’s papules, which are the markers of disease severity. Localised scleroderma includes morphea and linear scleroderma, where skin changes can lead to functional impairment and aesthetic concerns. In Sjogren syndrome, xerosis and vasculitis are common and can significantly impact patients’ comfort and disease management. By expanding our understanding of these specific cutaneous manifestations and their implications, we can better manage and treat these conditions, ultimately improving patients’ outcomes.

## 5. Conclusions

Understanding the specific cutaneous manifestations associated with each rheumatic disease allows for early diagnosis and tailored treatments, leading to improved outcomes and the better management of the disease. The early recognition of skin lesions facilitates timely intervention, optimizing patient care and disease prognosis. The holistic approach in assessing a patient, including all the systems and a urine dipstick for proteinuria, remains crucial in accurately diagnosing and managing these complex conditions.

## Figures and Tables

**Table 1:** Rheumatic diseases with initial cutaneous involvement in paediatric patients.

Rheumatic Diseases with Cutaneous Involvement	Cutaneous Manifestations
IgA vasculitis	Purpura in lower limbs and buttocks
Hypersensitivity Vasculitis	Skin nodules and ulcerations
Kawasaki disease	Polymorphic exanthema of the trunk; the erythema and oedema of palms and soles; the peeling of the borders of the fingernails and perineum; the erythema of the tongue, lips, and oropharynx; bilateral bulbar conjunctivitis; Beau lines
Behçet disease	Oral ulcers, genital ulcers, extragenital ulcerations, acneiform lesions, erythema nodosum
Polyarteritis Nodosa	Livedo reticularis, purpura, necrosis, and potential digital gangrene. Painful subcutaneous nodules along the affected vessels.
Takayasu Arteritis	Livedo reticularis, purpura, erythema nodosum, subcutaneous oedema, urticaria, digital gangrene, and ulcers are all possible skin manifestations of this condition.
ANCA-associated vasculitis	Palpable purpura and/or petechial rash. The less common skin findings include subcutaneous nodules, infarctions, livedo reticularis, Raynaud’s phenomenon, and subcutaneous swelling
Systemic lupus erythematosus	Malar rash, discoid rash, photosensitivity, oral ulcers, alopecia, purpura
Cutaneous lupus	Discoid lesions, erythematous plaques, photosensitivity, alopecia, hyper-de-pigmentation
Neonatal lupus	Photosensitive rash, periorbital erythema, annular lesions
Juvenile dermatomyositis	Heliotrope rash, Gotton’s papules, shawl sign, nailfold teleangectasias, calcinosis
Localised scleroderma	Morphea, linear scleroderma, atrophy, pigment changes
Sjogren syndrome	Xerosis, vasculitis, annular erythema, Raynaud’s phenomenon

## Abbreviations

KD: Kawasaki disease; IgAV (IgA vasculitis); SLE: systemic lupus erythematosus; DM: dermatomyositis; ARF: Acute Rheumatic Fever; JIA: juvenile idiopathic arthritis; AHEI: Acute Haemorrhagic Oedema of Infancy; CAAs: coronary artery aneurysms; BD: Behcet’s disease; PAN: Polyarteritis Nodosa; cPAN: cutaneous PAN; ANCAs: antineutrophil cytoplasmic antibodies; TA: Takayasu arteritis; ESR: erythrocyte sedimentation rate; CRP: C-reactive protein; ACE inhibitors: angiotensin-converting enzyme inhibitors; AAVs: ANCA-associated vasculitides; MPO: myeloperoxidase; PR3: proteinase 3; GPA: Granulomatosis with Polyangiitis; MPA: Microscopic Polyangiitis; EGPA: Eosinophilic Granulomatosis with Polyangiitis.
